# Evidence for Polymicrobic Flora Translocating in Peripheral Blood of HIV-Infected Patients with Poor Immune Response to Antiretroviral Therapy

**DOI:** 10.1371/journal.pone.0018580

**Published:** 2011-04-11

**Authors:** Esther Merlini, Francesca Bai, Giusi Maria Bellistrì, Camilla Tincati, Antonella d'Arminio Monforte, Giulia Marchetti

**Affiliations:** Department of Medicine, Surgery and Dentistry, Clinic of Infectious Diseases, “San Paolo” Hospital, University of Milan, Milan, Italy; University of Cape Town, South Africa

## Abstract

In advanced HIV infection, the homeostatic balance between gastrointestinal indigenous bacteria and gut immunity fails and microbes are able to overcome the intestinal barrier and gain the systemic circulation. Because microbial translocation is not fully controlled by antiviral therapy and is associated with inefficient CD4+ reconstitution, we investigated the profile of translocating bacteria in peripheral blood of 44 HIV-infected patients starting therapy with advanced CD4+ T-lymphopenia and displaying poor CD4+ recovery on virologically suppressive HAART. According to CD4+ reconstitution at 12-months HAART, patients were considered Partial Immunological Responders, PIRs (CD4+≥250/µl, n = 29) and Immunological non Responders, INRs (CD4+<200/µl, n = 15)). We show that PIRs and INRs present similarly elevated plasma levels of lipopolysaccharide (LPS) and its ligand sCD14 that were not lowered by virologically suppressive therapy. Bacterial 16S rRNA gene amplification and sequencing resulted in a highly polymicrobic peripheral blood microbiota both prior and after 12-month HAART. Several differences in bacterial composition were shown between patients' groups, mainly the lack of probiotic *Lactobacillaceae* both prior and after therapy in INRs. Failure to control microbial translocation on HAART is associated with a polymicrobic flora circulating in peripheral blood that is not substantially modified by therapy.

## Introduction

HIV infection causes dramatic damage to the gastrointestinal (GI) tract, that includes substantial disruption of gut microbiota composition with presence of microbes at higher pathogenic potential compared to less aggressive indigenous organisms, massive loss of gut-residing CD4+ T-cells, and down-regulation of GI tract genes expression [1], [2], [3].

Thus, a substantial breach of the anatomo-functional GI barrier occurs, with progressive failure of mucosal immunity and leakage into the systemic circulation of bacterial by-products, such as lipopolysaccharide (LPS) and bacterial DNA fragments, which contribute to systemic immune activation [1] [4], [5], [6].

Highly active antiretroviral therapy (HAART) only partially amends GI tract antomo-functional damage [1], [7] and intestinal microbiota, further hampering intestinal homeostasis [8] and sustaining microbial translocation [9], [10]. Thus, although circulating microbial products have been shown to decrease during HAART, they remain elevated, in turn affecting immune restoration [1], [4], [7].

In untreated HIV/AIDS, the highest degree of microbial translocation has been shown in patients with severe immune depression [1], [4]. Similarly, following HAART initiation, patients with blunted long-term CD4+ recovery show persistently elevated circulating LPS and bacterial DNA independently of HIV viremia reduction [1], [4], [11]. Moreover, elevated microbial translocation as well as failure to contain it in the course of virologically-suppressive HAART have been associated to an increased risk of HIV disease progression and death [7], [12].

Given the relationship between microbial translocation and CD4+ reconstitution, we asked whether HIV-infected patients with ongoing microbial translocation despite virologically effective HAART, maintain a polymicrobic flora translocating in peripheral blood possibly with an imbalanced ratio of harmless indigenous organisms to pathogenic species. Should this supposition be verified, the next question to be addressed would be whether systemic microbiota may affect immune reconstitution.

Thus, we aimed to investigate the composition of translocating microflora in peripheral blood of HIV-infected patients with poor CD4+ T-cell recovery on virologically-suppressive HAART. We specifically chose to focus on a group of HIV-infected patients starting therapy with severe immune depletion of ≤200 CD4+/µl, because the extent of CD4+ T-lymphopenia is inversely related to microbial translocation [1], [11], and is a major determinant of immune reconstitution on HAART [13], [14], [15].

## Results

### Patient characteristics

We prospectively studied 44 HIV-infected antiretroviral-naive patients starting HAART with severe CD4+ depletion (≤200 cells/µL). Patients' median (IQR) baseline CD4+ T-cell count was 63 (28–117)/µl. At 12 months of HAART, patients' median (IQR) CD4+ T-cell count was 281 (180–357)/µl.

At 12 months HAART, 29 (67%) were Partial Immunological Responders (PIRs, HIV-RNA<60 copies/mL; CD4+≥250/µl), and 15 (33%) were Immunological non Responders (INRs, HIV-RNA<60 copies/mL; CD4+<200/µl). Patients' demographics and clinical features at baseline and month 12 are shown in Table 1.

**Table 1:** Patients' characteristics.

CHARACTERISTICS	FRs (n = 29)	INRs (n = 15)	HIV- (n = 13)	p
**Age, years (IQR)**	40 (32–48)	36 (29–52)	27 (25–32)	.002
**Sex, Male**	21	13	2	.001
**Risk Factors**				
Eterosex	15	9	N/A	
Omosex	7	2		
IDU	3	1		
**Time since 1st HIV-Ab+, years (IQR)**	4 (2–6)	6 (1–7)	N/A	
**Duration HAART, months (IQR)**	30 (15–59)	28 (12–64)	N/A	
**HCV-Ab (yes)**	3	2	N/A	
**Previous AIDS diagnosis (yes)**	14	9	N/A	
**CD4+ T Cells (cells/µL) (n) (IQR)**			N/A	
Nadir	70a,b (40–140)	35^b^ (13–56)		
12 months after HAART	330^a^ (281–399)	166 (127–189)		
**Plasma HIV-1 RNA Log(cp/mL) (IQR)**			N/A	
Zenith	5,17^b^ (4,81–5,69)	4,78^b^ (4,16–5,16)		
12 months after HAART	1,7 (1,70–1,78)	1,7 (1,70–1,78)		
**Antiretroviral therapy, number**			N/A	
NRTI+PI	28	15		
NRTI+NNRTI	1	0		
**Microbial Translocation Markers**				
**sCD14 (µg/mL) (IQR)**				
Baseline	3,18 (2,7–4,5	3,07 (2,8–3,9)	1,96 (1,39–2,4)	.002
12 months after HAART	4,26 (2,5–8,5)	3,85 (2,8–11,6)		.001
**LPS(pg/mL) (IQR)**				
Baseline	75 (52–82	75 (51–85)	75 (75–79,41)	.567
12 months after HAART	75 (62–81)	75 (65–114)		.947

**NOTE:** Data are median (IQR -Interquartile range-). FRs: Full Responders; INRs : Immunological Non Responders; IDU: intravenous drug user; HAART: highly active antiretroviral therapy; NRTI: nucleoside reverse transcriptase inhibitor; NNRTI: non-nucleoside reverse transcriptase inhibitor; PI: protease inhibitor.

a p<.01 for FRs vs INRs;

b p<.01 for T0 vs T12.

### HIV+ patients starting HAART with severe CD4+ T-lymphopenia display persistently elevated microbial translocation

As expected, when compared to HIV-negative controls, HIV+ patients as a whole displayed significantly higher T0 and T12 sCD14 (HIV− 1,96 (1,9–2,3) µg/mL; HIV+, T0 3,07 (2,7–4,1) µg/mL, HIV+, T12 4,26 (2,8–8,5) µg/mL, p = .0001), confirming enhanced microbial translocation.

PIRs and INRs displayed comparable baseline circulating LPS: PIRs, 75 (52–82) pg/mL; INRs, 75 (51–85) pg/mL; p = .95, and sCD14: PIRs: 3,18 (2,7–4,5) µg/mL; INR: 3,07 (2,8–3,9) µg/mL; p = .73) (Table 1). Interestingly, by 12 months of HAART, no major changes in LPS and sCD14 levels were shown in both PIRs and INRs (LPS, T12: PIRs, 75 (62–139) pg/mL; INRs, 75 (65–114) pg/mL; p = .2 and p = .36 for T0 vs T12 in PIRs and INRs, respectively; sCD14, T12: PIRs, 4,26 (2,5–8,5) µg/mL; INRs, 3,85 (2,8–11,6) µg/mL; p = .1; p = .5 for T0 vs T12 in PIRs and INRs, respectively) (Table 1).

### INRs present increased circulating 16S rDNA levels

Because the finding of equally elevated circulating LPS despite divergent CD4+ recovery on virologically-suppressive HAART was somehow unexpected, we set out to further characterise microbial translocation by investigating bacterial DNA composition in plasma.

At baseline, 14/44 (32%) HIV+ patients yielded a positive 16S rRNA gene PCR amplification, with a significantly higher proportion of PCR-positive INRs (INRs: 8/15, 53%; PIRs: 6/29, 20%; p = .04). At T12, 7/44 (16%) HIV+ patients were PCR-positive, with a non significantly higher proportion of INRs: INRs: 4/15 (27%); PIRs: 3/29 (10%); p = .21. No PCR amplification was yielded in HIV-negative controls.

### HIV+ patients starting HAART with severe CD4+ T-lymphopenia display a polymicrobic circulating bacterial microbiota

Following amplification, 10 representative bacterial colonies/patient were sequenced. Thus: at T0, 8 PCR+ INRs made up to 80 sequenced bacterial colonies, and 6 PCR+ PIRs corresponded to 60 sequenced bacterial colonies; at T12, 4 PCR+ INRs made up to 40 sequenced bacterial colonies, and 3 PCR+ PIRs corresponded to 30 sequenced bacterial colonies.

**Figure 1:**
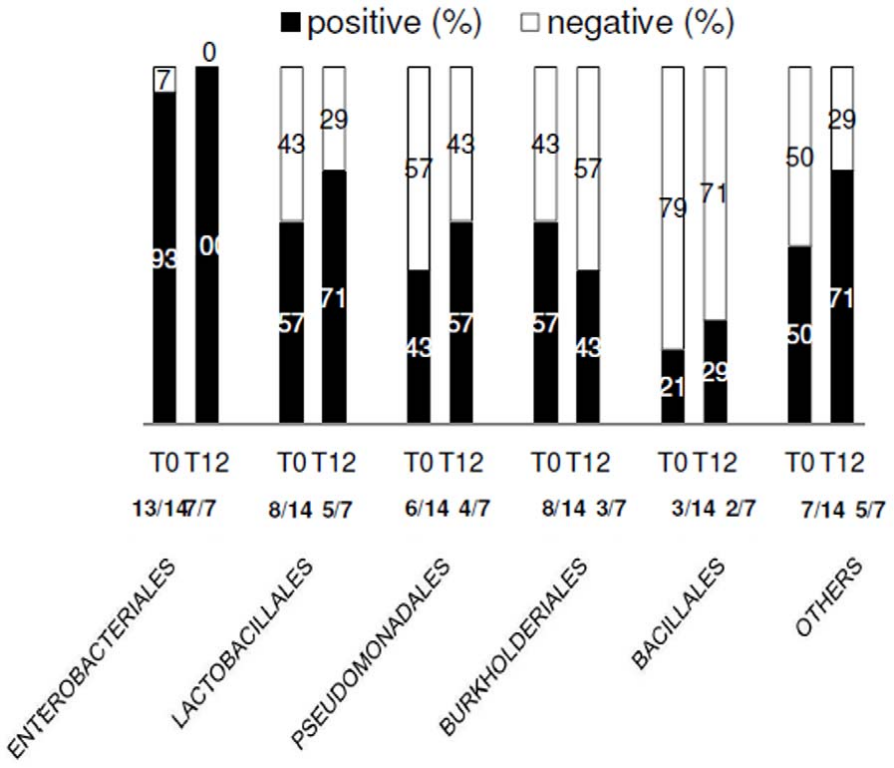
Characterization of translocating microflora in HIV+ antiretroviral naive patients. Patients' plasma samples were examined before and after 12 months of HAART for the presence and identification of DNA bacterial fragments using a broad-range 16S rRNA gene PCR amplification followed by sequencing analysis. At baseline, 14/44 (32%) HIV+ patients yielded a positive PCR amplification, whereas at T12, 7/44 (16%) HIV+ patients were PCR-positive. Sequencing analysis of HIV positive patients as a whole revealed multiple bacterial orders for each patient with no differences in the bacterial composition between baseline and T12. Positive (%) = # patients displaying a specific bacterial order/total # of PCR positive patients. Negative (%) = # patients negative for a specific bacterial order/total # of PCR positive patients.

Sequencing analysis of HIV positive patients as a whole revealed multiple bacterial orders for each patient with no differences in the bacterial composition between baseline and T12 (Figure 1). In particular, between T0 and T12, we found a similar proportion of patients displaying bacteria belonging to *Enterobacteriales* (T0: 13/14, 93%; T12: 7/7, 100% p = .75), *Lactobacillales* (T0: 8/14, 57%; T12: 5/7, 71% p = 1), *Pseudomonadales* (T0: 6/14, 43%; T12: 4/7, 57% p = 1), *Burkholderiales* (T0: 8/14, 57%; T12: 3/7, 43% p = .74) and *Bacillales* (T0: 3/14, 21%; T12: 2/7, 29% p = 1).

**Figure 2:**
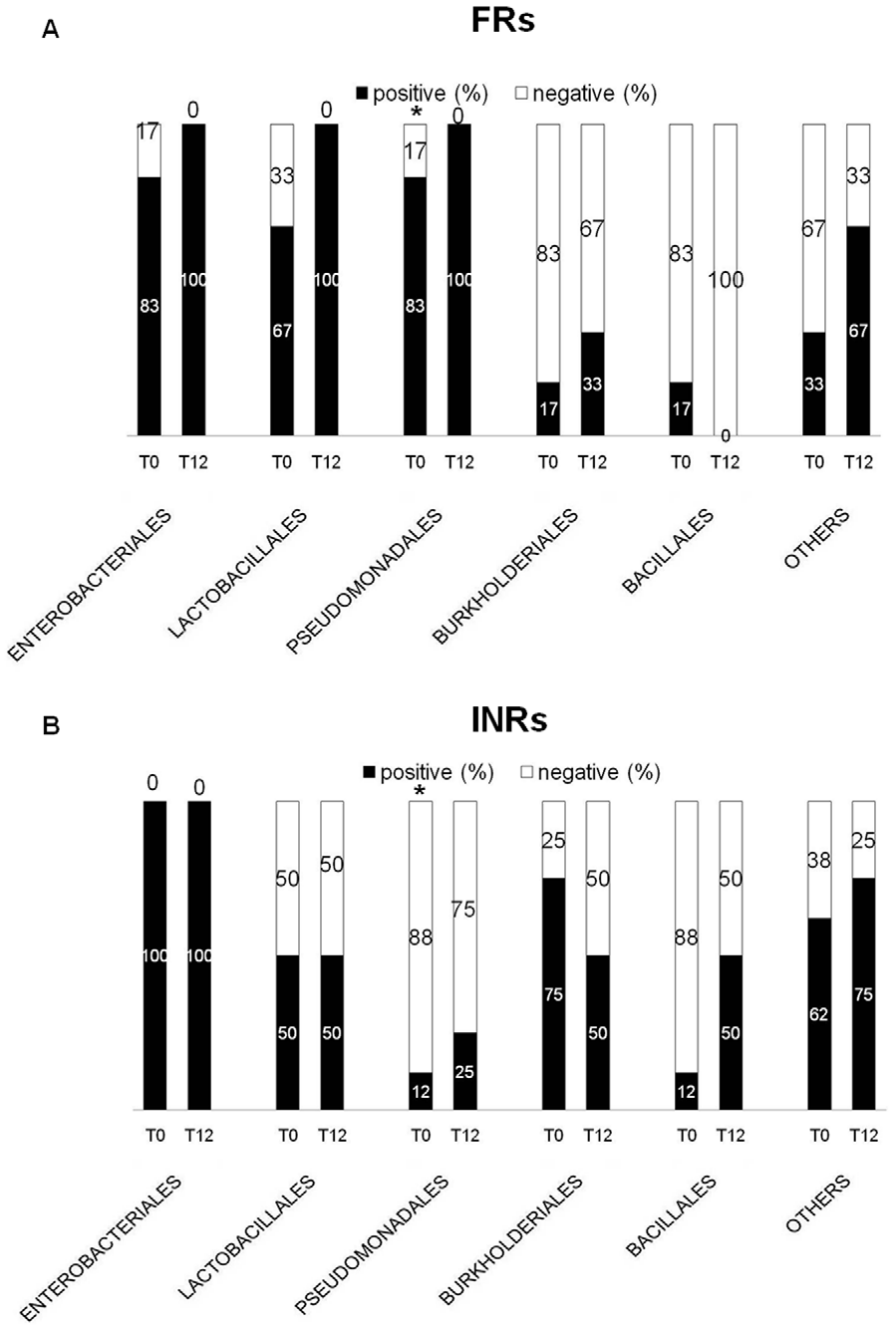
Characterization of translocating microflora in PIRs and INRs. Bacterial microflora translocating in peripheral blood was evaluated in 44 HAART-naive patients with severe immune depression (CD4+≤200/µL) at baseline and after 12 months of stable HAART (T12). At T12, 29 patients were partial immunological responders -PIRs- (CD4+≥250/µL; VL<60 cp/mL) and 15 were immunological-non-responders -INRs- (CD4+<200/µL; VL<60 cp/mL). At baseline, we found a significantly higher proportion of PCR-positive INRs (INRs: 8/15, 53%; PIRs: 6/29, 20%; p = .04). At T12 a non significantly higher proportion of INRs yielded a positive 16S gene PCR amplification: INRs: 4/15 (27%); PIRs: 3/29 (10%); p = .21. PIRs and INRs displaying a similar bacterial orders both at baseline and T12, except *Pseudomonadales* order *(FRs 5/6, 83% vs 1/8, 12% p = .026). Positive (%) = # patients displaying a specific bacterial order/total # of PCR positive PIRs (or INRs). Negative (%) = # patients negative for a specific bacterial order/total # of PCR positive PIRs (or INRs).

However, when we divided HIV+ patients in PIRs and INRs we found differences in bacterial composition between the two groups at baseline and T12 (Figure 2–4).

**Figure 3 pone-0018580-g003:**
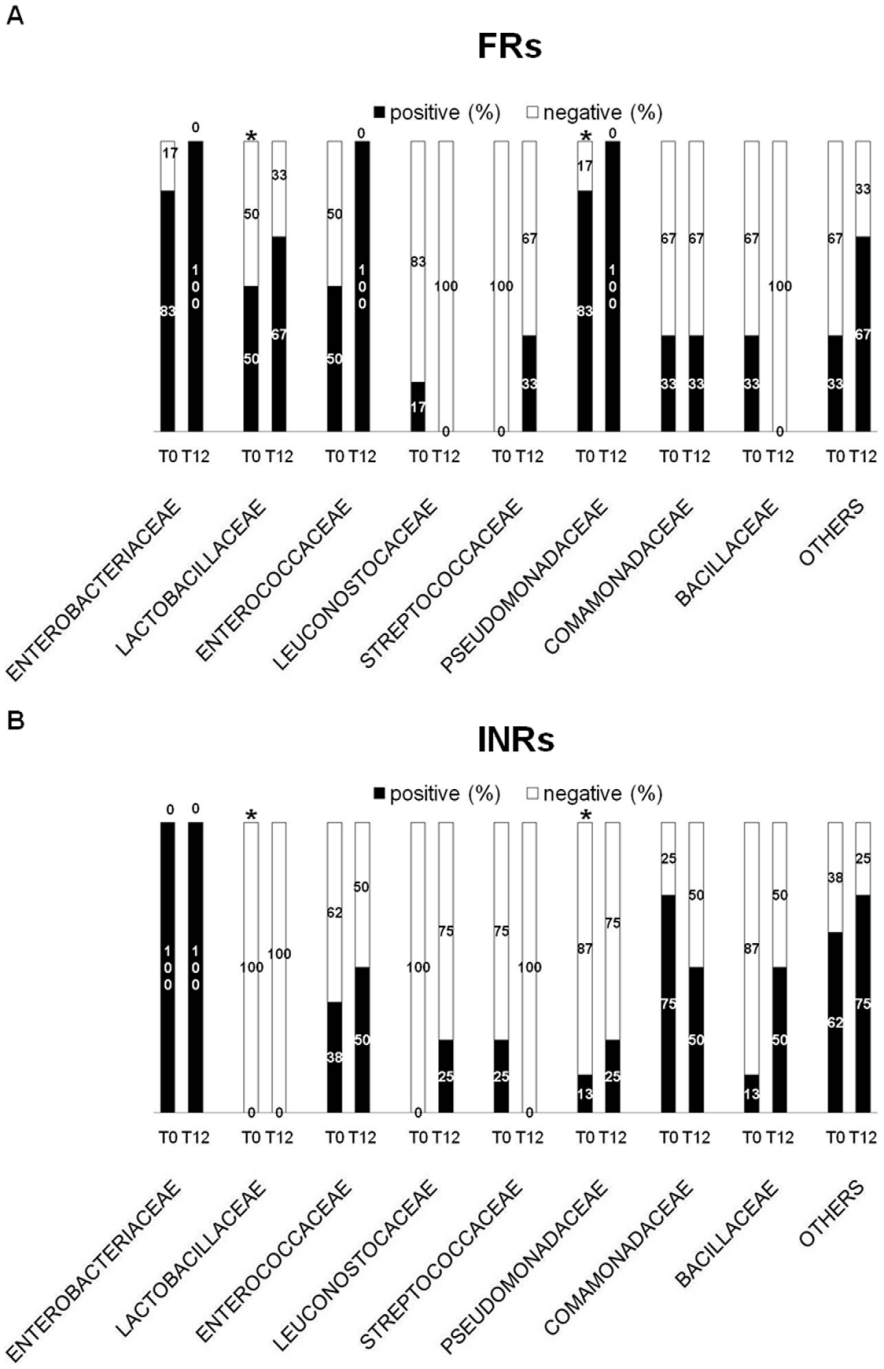
Identification of bacterial families in PIRs and INRs. Positive (%) = # patients displaying a specific bacterial family/total # of PCR positive PIRs (or INRs). Negative (%) = # patients negative for a specific bacterial family/total # of PCR positive PIRs (or INRs). PIRs and INRs presented a different profile in term of bacteria families. (**A**) PIRs displayed a similar composition of bacterial families between baseline and T12. (**B**) No major changes in bacterial families were seen in INRs after 12 months-HAART. At baseline, the significant differences between the two groups concerned *Lactobacillaceae* *(PIRs 3/6, 50% vs INRs 0/8 0%; p = .05) and Pseudomonadaceae *(PIRs 5/6, 83% vs INRs 1/8, 13%; p = .026). No differences between PIRs and INRs at T12.

**Figure 4:**
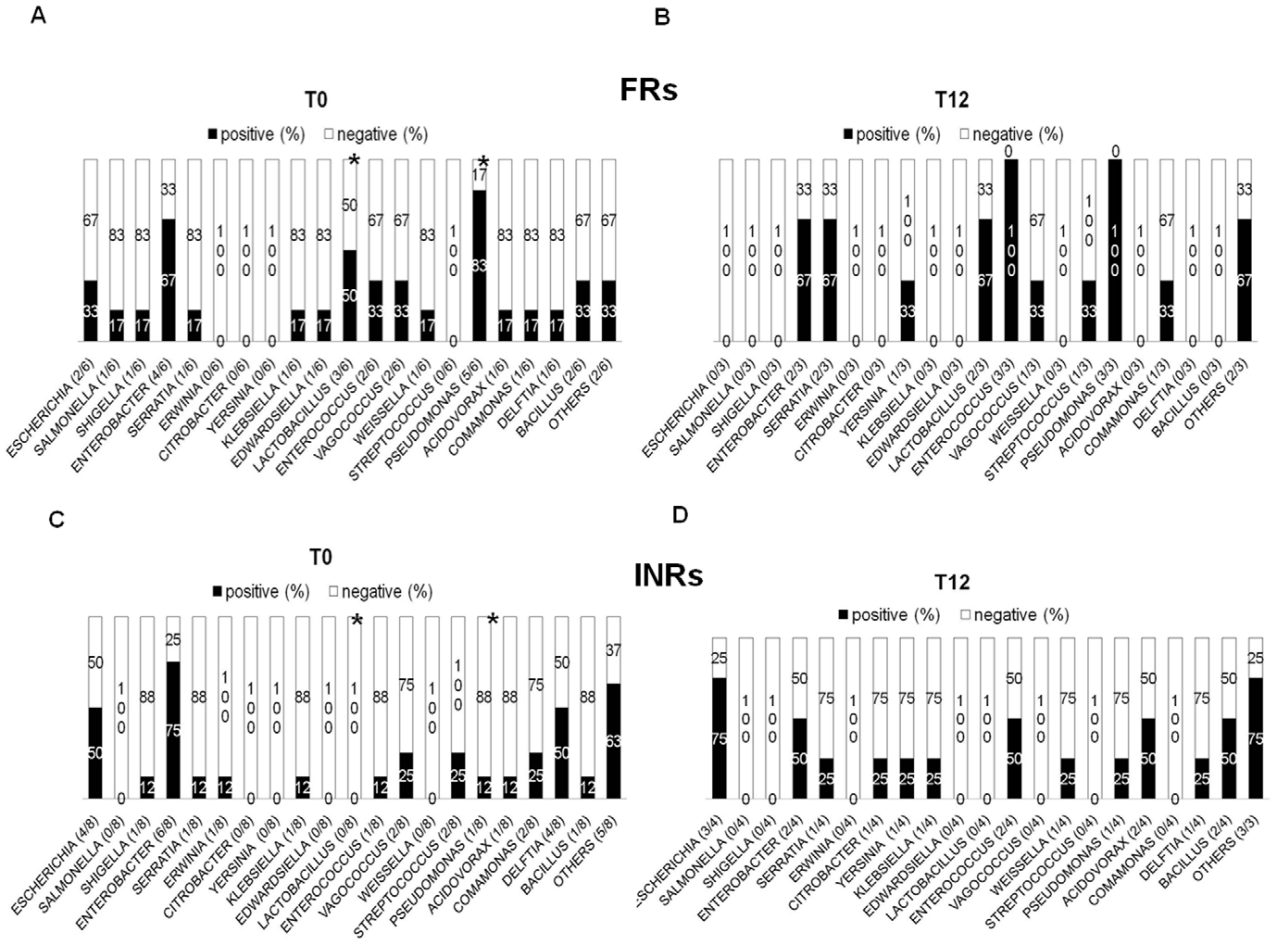
Bacterial genera identified in plasma sample of PIRs and INRs. Positive (%) = # patients displaying a specific bacterial genera/total # of PCR positive PIRs (or INRs). Negative (%) = # patients negative for a specific bacterial genera/total # of PCR positive PIRs (or INRs). (**A–B**) A similar composition of bacterial genera were seen in PIRs both at T0 and T12. (**C–D**) INRs presented no variation in bacterial genera following 12 months of HAART. At baseline, significant differences between PIRs and INRs were seen for *Lactobacillus sp* *(PIRs 3/6, 50% vs INRs 0/8 0%; p = .05) and Pseudomonas sp *(PIRs 5/6, 83% vs INRs 1/8, 13%; p = .026). No differences between the two groups at T12.

In terms of bacterial order, we found a similar baseline proportion of PIRs and INRs displaying bacteria belonging to *Enterobacteriales* (PIRs: 5/6, 83% vs INRs: 8/8, 100%, p = .43), *Lactobacillales* (PIRs: 4/6, 67% vs INRs: 4/8, 50%; p = .63), *Burkholderiales* (PIRs: 2/6, 17% vs INRs: 6/8, 75%; p = .28) and *Bacillales* (PIRs: 2/6, 17% vs INRs: 1/8, 12%; p = .54) (Figure 2A and B). A significantly higher proportion of PIRs displayed *Pseudomonadales* as compared to INRs (5/6, 83% vs 1/8, 12% p = .026) (Figure 2A and B).

No major changes in bacteria composition were shown following 12 months of HAART in both patients' groups (Figure 2A and B).

After identifying bacterial orders, we set out to investigate bacterial families (Figure 3A and B) and genera (Figure 4A and B). At baseline, a significantly higher proportion of PIRs displayed *Lactobacillaceae* (3/6, 50% vs 0/8 0%; p = .05) and *Pseudomonadaceae* (5/6, 83% vs 1/8, 13%; p = .026) as compared to INRs (Figure 3A and B). In particular, the differences concerned *Lactobacillus sp* (PIRs 3/6, 50% vs INRs 0/8 0%; p = .05) and *Pseudomonas sp* 5/6, 83% vs 1/8, 13%; p = .026) (Figure 4A and C).

After 12 of months HAART in both patients' groups, no significant changes were shown in bacteria families (Figure 3A and B) and genera (Figure 4B and D).

## Discussion

Through the joint quantification of gut-derived bacterial macromolecules and genotypes, we hereby assessed the quality of microbial translocation in peripheral blood of HIV-infected patients starting HAART with severe immune depression and lacking CD4+ recovery on therapy, and have made the following observations. First, severely immune depressed HIV-infected patients fail to efficiently control translocation of microbial macromolecules following HAART initiation. Second, HIV-infected patients maintaining heightened microbial translocation on virologically-suppressive HAART display a circulating microbiota that is polymicrobic at the genotype level and that is not substantially modified by therapy.

Both LPS/sCD14 plasma levels have been described to decrease following HAART initiation [1], [4], [11], [16]. However, while patients starting HAART with high CD4+ counts present a substantial LPS/sCD14 plasma reduction that is inversely correlated with the magnitude of CD4+ recovery [1], [4], a less efficient containment of microbial translocation has been shown in naïve patients starting therapy with low CD4+ [11], [16]. Accordingly, we hereby describe that patients with severe CD4+ T-lymphopenia at HAART initiation maintain heightened circulating LPS/sCD14 after 12 months of therapy despite HIV-viremia control.

A possible explanation of the less efficient control over microbial translocation in this patients' cohort might be consistent with the dramatic structural damage of the intestinal barrier described in untreated advanced HIV/SIV infection [9], [17], with ever-increasing content of microbial by-products infiltrating intestinal *lamina propria* and gaining the circulation. The GI tract damage and occurring microbial translocation might be hardly repaired by the late institution of therapy, in turn favouring the continuous passage into the systemic circulation of a highly polymicrobic intestinal flora. Furthermore, in these patients a delayed decrease in sCD14/LPS plasma levels following HAART initiation.

Increased levels of circulating microbial by-products and markers of immune response to microbial translocation have been associated to increased HIV disease progression and mortality even in the context of continuous antiviral therapy and independently of CD4+ count and HIV-viremia [7], [12], advocating a role of LPS/sCD14 in predicting CD4+ reconstitution and/or risk of clinical progression on HAART. However, by showing that following 12 months of virologically-suppressive HAART, HIV-infected patients with advanced immune depression fail to control microbial translocation and microbial-driven monocyte activation and maintain elevated free LPS/sCD14, our findings seem to suggest that the quantification of LPS/sCD14 may not be sufficient to predict immunological failure to HAART in these patients. Research directly comparing sCD14/LPS levels changes on HAART in patients with different CD4+ counts at HAART start as well as longer patients' follow-up are needed to further confirm such hypothesis.

The main finding of our research is that HIV-infected patients starting therapy with severe CD4+ depletion display a circulating microbiota that is highly polymicrobic at the genotype level and that is not substantially modified by therapy.

Interestingly, more than 90% of HIV-infected patients harbour a bacterial population enriched with *Enterobacteriales*, whereas less than 60% display the probiotic *Lactobacilllales*, with the same proportion being maintained on virologically-suppressive HAART.

Interestingly, despite equal circulating LPS, we observed some differences in the composition of translocating microflora in patients with different extent of CD4+ T-cell recovery on virologically suppressive HAART.

In particular, in INRs we describe the presence of the more potentially pathogenic *Enterobacteriaceae* with no evidence of *Lactobacillus spp.*. *Lactobacillus spp.* possess immunomodulatory and anti-inflammatory properties [18], including suppression of pro-inflammatory cytokines production from *E.coli* LPS-activated monocytes [19]. This might result in disproportionate antigenic challenge that is not contained by counter-regulatory bacterial-derived factors, overall exacerbating immune activation.

Despite our study was not designed to identify statistically significant associations between peripheral blood microbial genotypes and immunological outcome, these findings highlight the potential correlations between the bacterial species in the systemic circulation and immune reconstitution. Thus, our research should be used to generate hypotheses to be tested in larger studies aimed at investigating the role of translocating bacteria in the regulation of mucosal and systemic immune homeostasis, and the association with HAART-driven immune reconstitution.

From a mechanistic standpoint, our research detail a polymicrobic translocating flora including pathogenic and symbiotic bacteria in severely immune-depressed HIV+ patients, which is not seen in HIV-negative subjects, and is not reverted by virologically-suppressive therapy, suggesting substantial failure of gut immunity in controlling bacteria translocation [1], [2]. Several concurring mechanisms likely contribute to such polymicrobic translocating flora, comprising impairment of resident microflora, macrophage dysfunctions, persistent gut epithelia barrier damage or loss of GI tract Th17 cells [6], [20], [21].

Our study has several limitations. The PCR, cloning and sequencing techniques that we used are somehow outdated and have been replaced by more modern techniques such as pyrosequencing [22] [23]. Potential biases of our experimental approach include more efficient amplification of certain bacteria *versus* others, and low sensitivity of the assay.

A further limitation is the number of colonies analysed per patient. Increasing the number of colonies sequenced per patient as well as the number of patients studied overall would certainly give a deepest/adequate representation of the microbiota present in peripheral blood of HIV-infected patients.

However, the identification of statistically relevant associations between peripheral blood microbial genotypes and immune recovery was beyond the purpose of our study. Indeed, the main finding of our research is that HIV-infected patients starting therapy with severe CD4+ depletion display a circulating microbiota that is highly polymicrobic at the genotype level and that is not substantially modified by therapy: these findings are already evident by sequencing 10 colonies/patient.

Although the conclusions of our study are based on a limited number of patients and thus very preliminary, this is to our knowledge the first report to suggest polymicrobial translocation of gut flora in HIV-infection. Further research is needed in patients with different CD4+ counts at HAART start as well as longer patients' follow-up to further confirm such hypotheses.

This might also provide a rational basis for the investigation of adjuvant therapeutical approaches aimed at recovering the symbiotic host-microorganism relationships, in turn containing chronic systemic inflammation and contributing to a more efficient immune reconstitution on HAART [24].

## Methods

### Patients

We longitudinally investigated 44 HIV-infected antiretroviral-naive patients, starting HAART with severe CD4+ depletion of ≤200 cells/µL. After 12 months of stable HAART, we divided HIV-infected individuals into two groups according to viro-immunological response to therapy: (i) Partial Immunological Responders -PIRs, HIV-RNA<60 copies/mL and CD4+ count ≥250/µl in at least three consecutive determinations; (ii) Immunological non Responders -INRs, HIV-RNA<60 copies/mL and CD4+ count <200/µl in at least three consecutive determinations. Exclusion criteria were initiation of therapy during primary HIV infection, signs or symptoms of gastrointestinal disease and chirrosis.

As controls, we studied plasma samples from 13 HIV-negative healthy subjects.

All enrolled patients provided written informed consent according to the Ethical Committee of our Institution (Comitato Etico, Ospedale “San Paolo” and Comitato Etico, Milan, Italy). The ethics committee (Comitato Etico, Ospedale “San Paolo” and Comitato Etico, Milan, Italy) specifically approved this study.

### Plasma LPS and sCD14

Microbial translocation was evaluated by plasma levels of lipopolysaccharide (LPS) (LAL test; Kinetic-QCL; Bio Whittaker, Walkersville, MD, USA) and sCD14 (ELISA assay, R&D, Italy), according to manufacturer's protocols.

### Broad-range PCR for measurement of bacterial 16S rRNA gene

Bacterial DNA detection was performed using a broad-range 16S rRNA gene PCR amplification followed by sequencing analysis in all 44 HIV+ patients and 13 HIV− controls. Following DNA extraction, PCR amplification was performed as previously described [25], with the following thermal cycler conditions: 94°C for 5 min, 40 times for 1 minute each time at 94°C, 55°C and 72°C, and 10 min at 72°C. To confirm DNA integrity, we also amplified the housekeeping gene beta-globin. Standard PCR precautions were taken to avoid samples cross-contaminations.

### Cloning and Sequencing of PCR products

The 360-bp PCR products were cloned into the pCR®2.1 -TOPO® Vector (Invitrogen, San Diego, California, USA.) and 10 representative bacterial colonies per patient were sequenced (ABI®PRISM 3130XL Genetic Analyzer, Applied Biosystem, Foster City, CA, USA). Only 98–100% homologies by “Basic Local Alignment Search Tool” (BLAST) were considered valid.

### Statistical analysis

All continuous variables were presented as median ± interquartile ranges (25^th^–75^th^ percentile), while categorical data were shown as absolute numbers and percentages. Quantitative values were tested for normality with Shapiro-Wilk test and all the analyzed variables deviated from normality. Pearson's chi square or Fisher's exact tests were used to evaluate associations of the categorical variables between HIV− healthy subjects and HIV+ patients or among HIV− healthy subjects, PIRs and INRs. The Mann Whitney U test with the Montecarlo method for small samples was used for the comparison of continuous parameters between the 2 groups, while eventual differences among the three groups of patients were explored with Kruskal-Wallis test. Results of bacterial sequencing analysis are presented as # of patients displaying a specific bacterial order, family, species/total # of PCR-positive patients. To compare the percentages of PCR positive-negative between INRs and PIRs and between different time points (T0 and T12) in the same group of patients, we used Pearson's chi square or Fisher's exact tests. The same statistic test was used to analyze eventual differences in bacterial composition (orders, families and species) between PIRs and INRs and between T0 and T12. A value of p<0.05 was considered denoting statistical significance. Statistics were performed using SPSS software (version 17.01; SPSS).
